# Piezoelectric and Magnetically Responsive Biodegradable Composites with Tailored Porous Morphology for Biotechnological Applications

**DOI:** 10.1021/acsapm.2c01114

**Published:** 2022-11-09

**Authors:** Teresa Marques-Almeida, Vitor Correia, Eduardo Fernández Martín, Ander García Díez, Clarisse Ribeiro, Senentxu Lanceros-Mendez

**Affiliations:** † Physics Centre of Minho and Porto Universities (CF-UM-UP), University of Minho, Braga4710-057, Portugal; ‡ LaPMET - Laboratory of Physics for Materials and Emergent Technologies, University of Minho, Braga4710-057, Portugal; § CMEMS − UMinho, University of Minho, Guimarães4800-058, Portugal; ∥ LABBELS − Associate Laboratory, Braga, Guimarães4800-058, Portugal; ⊥ BCMaterials, Basque Centre for Materials and Applications, UPV/EHU Science Park, Leioa48940, Spain; # IKERBASQUE, Basque Foundation for Science, Bilbao48009, Spain

**Keywords:** poly(3-hydroxybutyric acid-*co*-3-hydroxyvaleric acid), magnetostrictive iron oxide, magnetically responsive composites, porosity, biomedical applications, mechano-electrical stimuli

## Abstract

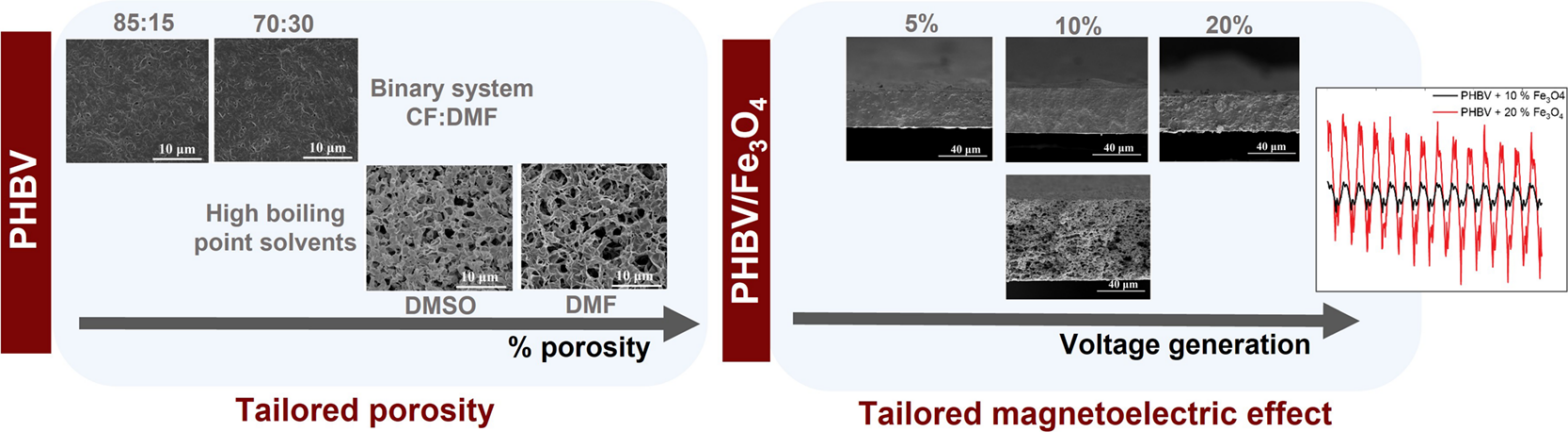

The biomedical area in the scope of tissue regeneration pursues the development of advanced materials that can target biomimetic approaches and, ideally, have an active role in the environment they are placed in. This active role can be related to or driven by morphological, mechanical, electrical, or magnetic stimuli, among others. This work reports on the development of active biomaterials based on poly(3-hydroxybutyric acid-*co*-3-hydroxyvaleric acid), PHBV, a piezoelectric and biodegradable polymer, for tissue regeneration application by tailoring its morphology and functional response. PHBV films with different porosities were obtained using the solvent casting method, resorting to high-boiling-point solvents, as *N*,*N*-dimethylformamide (DMF) and dimethylsulfoxide (DMSO), and the combination of chloroform (CF) and DMF for polymer dissolution. Furthermore, magnetoelectric biomaterials were obtained through the combination of the piezoelectric PHBV with magnetostrictive iron oxide (Fe_3_O_4_) nanoparticles. Independently of the morphology or filler content, all biomaterials proved to be suitable for biomedical applications.

## 1 Introduction

Natural-based biomaterials are an increasing trend in the biomedical field. Their benefits compared to synthetic materials are increased biocompatibility, biodegradability, remodeling, and biologically inert behavior with respect to inflammatory episodes, making them more pursued in the biomedical industry and tissue regeneration applications.^1^ Frequently, the selected materials for tissue engineering applications are required to target specific biomimetic approaches and ideally have an active role in the environment where they are positioned.^2−4^ In this context, electroactive materials, namely, with piezoelectric characteristics, are gaining general interest in the biomedical area for the development of biomedical devices as medical sensors and actuators.^5−7^ Nevertheless, the most recent uses are in the area of tissue engineering as scaffolds^8,9^ and drug delivery systems.^10,11^ Piezoelectricity, in fact, occurs in a variety of human tissues, including bones, skin, cartilage, ligaments, and tendons, as well as in DNA,^12^ so biomaterials that have this property are relevant to mimic specific microenvironments with electrical and mechano-electrical cues.^13,14^ Their active behavior allows to translate a surface mechanical stimulus in a dynamic process into electrical potential and vice versa that support tissue regeneration and function recovery.^15^ Up to now, most efforts on piezoelectric driven tissue engineering rely on poly(vinylidene fluoride) (PVDF), a biocompatible but nondegradable polymer.^12^ Nevertheless, in some context, it is relevant the possibility to implement piezoelectric biodegradable polymer with suitable piezoelectric response that can biodegrade during the regeneration process. Poly(3-hydroxybutyric acid-*co*-3-hydroxyvaleric acid) (PHBV) (a copolymer of polyhydroxybutyrate (PHB) from the polyhydroxyalkanoates (PHA) family) is a thermoplastic aliphatic polyester, derived from bacterial polymerization, that fills these requirements, and it is also a biocompatible and biodegradable natural polymer. It presents a piezoelectric response of 0.7–1.3 pC N^–1^,^916,17^ lower than the one of PVDF (24–34 pC N^–1^) but suitable for applications in the biomedical area, such as cell stimulation.^18^ Furthermore, it is easily processed, allowing the development of different morphology matrices, as dense and porous films, fibers, 3D scaffolds, and microspheres, relying on different processing techniques.^16^ The ability to be processed under different morphologies makes PHBV suitable to be tailored for specific microenvironments and applications, such as drug delivery,^19,20^ tissue engineering,^16,21^ antibacterial substrates,^22^ and packaging,^23^ among others. Porous matrices have been required to mimic porous structures in the human body as the cancellous bone morphology for example. Porous interconnectivity can influence cellular dynamics and facilitate cell attachment, elongation and proliferation, nutrient diffusion, and vascularization.^24,25^ Moreover, as a drug carrier, the porous structure enables an increase in the drug loading capacity and a controlled release of the drug.^26,27^

Particularly interesting in this context is the combination of materials and magnetostrictive nanoparticles, allowing to develop magnetically responsive platforms, including magnetomechanical (for nonpiezoelectrically responsive samples) and magnetoelectric (for piezoelectrically responsive samples) platforms. Those materials are able to mechanically and/or electrically stimulate the cells upon magnetic solicitation,^15,28^ which is particularly interesting for a variety of sensing and actuation applications.^29^ Fe_3_O_4_ magnetic nanoparticles have been widely studied because of their high coercivity, low Curie temperature, and magnetostrictive and superparamagnetic properties.^30^ Furthermore, their nontoxic behavior and biocompatibility allows its use in biomedical applications that require a biodegradable polymer.^31^

In this work, a versatile biocompatible active material platform for tissue engineering applications consisting of biodegradable and piezoelectric polymer PHBV is demonstrated, without and with Fe_3_O_4_ nanoparticles, processed in the form of dense and porous morphologies by solvent casting with high-boiling-temperature solvents, *N*,*N*-dimethylformamide (DMF) and dimethylsulfoxide (DMSO), and a binary solvent system including chloroform (CF) and DMF. To the best of our knowledge, this is the first work that report on porous PHBV films and their interaction with magnetostrictive nanoparticles. It not only addresses the formation of pores but also variations of the degree of porosity, allowing the development of an active material platforms adaptable to a wide range of biomedical applications.

## 2 Materials and Methods

### 2.1 Materials

Pure PHBV powder (99%) (*M*_w_ = 4.7 × 10^6^ g mol^–1^) with a 3% hydroxyvalerate (HV) (mol/mol) was purchased from Natureplast and Fe_3_O_4_ nanoparticles (30 nm diameter) from NanoAmor. CF with 99% purity was supplied from Fisher, DMF with pure grade from Honeywell, and DMSO from Sigma-Aldrich. All materials were used as received from the provider.

### 2.2 Processing of the Porous PHBV and PHBV/Fe_3_O_4_ Films

Based on a previous study,^16^ all PHBV films were developed with a polymer concentration of 10% (w/v). For porous films, the PHBV powder was dissolved in DMF (PHBV_DMF) or in DMSO (PHBV_DMSO) under magnetic stirring at 120 °C for 2 h. For the porous films obtained through binary solvent systems, two different CF–DMF ratios were evaluated: 70:30 (v/v) (PHBV_70:30 (CF:DMF)) and 85:15 (v/v) (PHBV_85:15 (CF:DMF)). For that, PHBV powder was first dissolved in CF under magnetic stirring at 50 °C for 1 h, and then, the correspondent volume of DMF was added and magnetically stirred for 15 min.

Nonporous composite films of PHBV and Fe_3_O_4_ were developed with a polymer concentration of 10% (w/v) in CF and nanoparticle concentrations of 5, 10, and 20% (w/w). To avoid nanoparticle agglomeration and ensure good dispersion, Fe_3_O_4_ nanoparticles were first dispersed in CF within an ultrasound bath for 1 h. PHBV powder was then added to the solution and magnetically stirred at 50 °C for 1 h until complete polymer dissolution. PHBV dense films without nanoparticles were also processed, and for that, PHBV powder was dissolved in CF under magnetic stirring at 50 °C for 1 h.

A composite porous film with 10% (w/w) Fe_3_O_4_ nanoparticles was also prepared based on the binary solvent system of CF–DMF in an 85:15 (v/v) relation. After nanoparticle dispersion in the corresponding CF volume, PHBV and the respective volume of DMF were added to obtain the 10% w/v concentration and maintaining the 85:15 (v/v) proportion of CF/DMF. The solution was also magnetically stirred at 50 °C for 1 h until complete dissolution.

After dissolution, all films were obtained by the solvent casting method. Each prepared solution was spread on a clean glass substrate, followed by solvent evaporation at RT. Solvent evaporation required 3 days for porous films and 20 min for dense films. For better understanding, films nomenclature is presented in Table 1.

**Table 1:** Respective Nomenclature for Processed PHBV Films^a^

	solvents	solvents concentration	filler %	nomenclature
porous films	CF:DMF	70:30	NA	PHBV_70:30 (CF:DMF)
		85:15	NA	PHBV_85:15 (CF:DMF)
			10	PHBV_10%Fe_3_O_4_ (CF:DMF)
	DMF	100	NA	PHBV_DMF
	DMSO	100	NA	PHBV_DMSO
dense films	CF	100	NA	PHBV
			5	PHBV_5%Fe_3_O_4_
			10	PHBV_10%Fe_3_O_4_
			20	PHBV_20%Fe_3_O_4_

a “NA” stands for nonapplicable.

### 2.3 Morphological, Chemical, and Thermal Property Characterization

Film morphology was assessed by scanning electron microscopy (SEM). The samples were coated with a gold thin layer in a Polaron sputter (model SC502) and visualized by SEM with a 5 kV electron beam acceleration in an FEI Nova 200 from the NanoSEM system. Regarding the composite films, energy dispersive spectroscopy (EDS) was performed in a Pegasus X4M (EDS-EBSD) system from EDAX.

The thickness of the films was measured using a digital micrometer Dualscope 603–478 from Fischer.

The overall porosity (ε) of the porous films was measured by the liquid displacement method, using a pycnometer and eq 1:
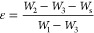
1where *W*_1_ corresponds to the weight of the pycnometer filled with absolute ethanol and *W*_s_ to the weight of the sample tested. The sample was immersed in the pycnometer and, after its saturation, ethanol was added to complete the volume of the pycnometer. *W*_2_ refers to the weight of the system. Finally, the sample was removed from the pycnometer to obtain *W*_3_. Three replicates of each sample were analyzed to obtain the mean porosity of the corresponding processed film. All results are presented as mean ± standard deviation.

Fourier-transform infrared spectroscopy (FTIR), differential scanning calorimetry (DSC), thermogravimetric analysis (TGA), and contact angle measurements were carried out to assess chemical and thermal properties of the materials. FTIR measurements were performed in an Alpha II-Platinum ATR/Transmission (Bruker) apparatus in attenuated total reflectance (ATR) mode, through 64 scans ranging from 4000 to 600 cm^–1^. For DSC, approximately 3 mg of each material was placed into aluminum pans of 50 μL and analyzed in a DSC 6000 apparatus (Perkin-Elmer). Samples were heated from 30 to 200 °C at a 10 °C min^–1^ rate. Regarding TGA, approximately 10 mg of each sample was placed on a TGA 4000 apparatus (Perkin-Elmer) and measured from 30 to 500 °C at 20 °C min^–1^.

The wettability of the samples was evaluated by contact angle measurements in six replicates for each sample using a Data Physics OCA20, establishing the correspondent mean and standard deviation.

### 2.4 Magnetic and Mechanic Characterization

The mechanical properties of the films were evaluated at room temperature by stress–strain tests at a 100 μm s^–1^ deformation speed in a Linkam LNP95 apparatus with a modular force stage with a loading cell of 10 N. Mechanical tests were carried on rectangular (25 × 10 mm, thickness ranging from 28 to 102 μm) samples in triplicate. Young’s modulus was determined in the linear range of elasticity (between 0 and 0.3%) and estimated from the average of triplicate’s measurements.

The magnetic response of the developed PHBV/Fe_3_O_4_ films was evaluated at room temperature by vibrating-sample magnetometry (MicroSense EZ7 equipment) with a maximum field of 10 kOe.

### 2.5 Corona Poling and Microscopic Morphological Features and Piezoelectric Response

PHBV neat films were poled in a custom-made corona discharge chamber to optimize the piezoelectric response. After an optimization procedure, PHBV samples were poled after 2 h of corona poling at 160 °C with a 10 kV applied voltage.

The morphology and the microscopic piezoelectric response of poled and nonpoled PHBV films was carried out using piezoresponse force microscopy (PFM).^32^ This characterization method uses standard scanning force microscopy (SFM) operated in contact mode, using a conducting tip. The tip induces deformations on the sample that lead to periodic vibrations in it, which are transmitted to the tip and read through a lock-in amplifier.^33^ This allows the simultaneous characterization of the topography and the piezo response amplitude and phase, indicating the possible orientation of the ferroelectric domains. Furthermore, properties such as polarization reversal and hysteresis can be studied at specific regions of the samples.^34^ For PFM measurements, a nano-observer atomic force microscope (AFM) from Concept Scientific Instruments (CSI, France), equipped with a high voltage amplifier, was used in piezo response mode at room temperature in air. The used tips were Pt/Ir-coated N-type Si tips (AppNano ANSCM series), with a nominal spring constant of 3 N m^–1^, an intrinsic resonance frequency of 60 kHz, and a tip radius of 30 nm. After calibration with the spectroscopy curve, the voltage set point was selected so that the force exerted on the sample was around 10 nN, enough for a soft polymeric material and low enough to avoid damaging of the tip.^35^ The AC voltage applied to the tip had an amplitude of 4 V. To extract the topography images, an area of 10 μm^2^ was scanned, with a resolution of 256 × 256 points at a scan rate of 1 Hz. To characterize the switching behavior, an additional DC voltage was applied to the tip, varying from −100 to 100 V, while simultaneously measuring the piezoelectric response. The images obtained from the microscope were treated with the Open-Source software Gwyddion.

### 2.6 Magnetomechanical Response

Regarding biomedical applications and in order to characterize the magnetic stimuli that the developed composite films would produce in a magnetic cultivation system in vitro,^15^ the magnetomechanical characterization of the samples was performed at the same height as the height of cultivation, in relation to the stimulation magnet table.

First, the magnetic characterization of the magnetic table was performed using a Hirst GM08 gaussmeter, with a transverse probe (PT8029), which allows measuring AC and DC fields with a maximum amplitude of 3 T and a resolution of 1 mT, using an USB/RS232 connection, to record the acquired data. To ensure the correct measurement of the generated field, concentric measurements with the center of the cultivation wells were performed by displacing the measuring tip, coupled to a graduated X-Y table, along the two axes. After the magnetic component was analyzed, the deformation of the sample was characterized under magnetic stimulation. For that purpose, a strain gauge (L2A-06-062LW-120, Vishay Precision Group, Inc) was attached to the sample using a LOCTITE Hysol 3425 two-component araldite glue. The sensor variation measurement is based on the need to obtain a relatively high sampling rate, considering the bioreactor operating at a frequency of 0.6 Hz (40 samples per second), and high resolution, considering the low deformation of the sample. Thus, resistance variation was measured by connecting the sensor terminals directly to Rigol DM3068 6 1/2 digital multimeter, operating in relative variation mode that correspond to an accuracy above 3 mΩ. The multimeter was connected via USB to the computer control application, developed in C++ for control and data recording.

### 2.7 Cell Culture

MC3T3-E1 preosteoblasts cells obtained from Riken Bank were used in cytotoxicity and adhesion tests. Cells were grown in Dulbecco’s modified Eagle’s medium (DMEM, Biochrom) containing 4.5 g L^–1^ glucose supplemented with 10% of fetal bovine serum (FBS, Biochrom) and 1% of penicillin/streptomycin (P/S, Biochrom). Cells were seeded and incubated in 75 cm^2^ culture flasks at 37 °C under humidified air and a 5% CO_2_ atmosphere. Growth medium was changed every two days, and cells that reached around 60–70% confluence were detached with 0.05% trypsin–ethylenediaminetetraacetic acid (trypsin–EDTA, Biochrom).

### 2.8 Cytotoxicity Evaluation

An indirect cytotoxicity assay was carried out to assess the sample cytotoxicity, following the general guidelines provided by the ISO 10993-5 standard test method. Cell viability was obtained through a 3-(4,5-dimethylthiazol-2-yl)-2,5-diphenyltetrazolium bromide (MTT) assay.

Porous and dense pristine PHBV and composite films were cut into 13 mm diameter discs, sterilized by ultraviolet light, for a duration of 30 min on each side, and washed three times in a phosphate buffer saline (PBS) solution. Then, four replicates of each sample were placed in a 24-well tissue culture polystyrene plate, covered with DMEM (Biochrom, Berlin, Germany) containing 4.5 g L^–1^ glucose supplemented with 10% FBS and 1% P/S, and incubated at 37 °C in 95% humidified air containing 5% CO_2_ during 24 h. At the same time, preosteoblast cells (MC3T3-E1 cell line, Riken bank) were seeded at a density of 3 × 10^4^ cell mL^–1^ in a 96-well tissue culture polystyrene plate and incubated for 24 h in DMEM to ensure cell attachment to the plate. After 24 h, the cell medium was removed and replaced by the medium in contact with the different samples (100 μL per well). Furthermore, negative and positive controls were measured for cell viability: cells in fresh DMEM for negative control and cells in contact with a 20% DMSO in DMEM solution for positive control. After 72 h of incubation, the indirect cell viability was assessed by the MTT assay, replacing the medium by a 10% MTT solution in DMEM, in contact for 3 h. After the incubation time, MTT formed crystals were dissolved in DMSO and the optical density was measured at 570 nm in a microplate reader (Biotech Synergy HT). Cell viability was then calculated according to eq 2.^16^
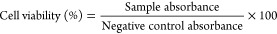
2

### 2.9 Cell Adhesion

Cells were cultured for 24 h on porous and dense pristine PHBV and composite films surfaces to study cell adhesion. Three replicates of sterilized 10 mm discs of each film were cut and placed in a 48-well tissue culture polystyrene plate. Preosteoblasts cell suspension was prepared and seeded 1 × 10^4^ cells on each sample well and incubated for 24 h under the same conditions as for initial cell culture. After this time, cells were fixed with 4% paraformaldehyde for 10 min at 37 °C and washed with PBS 1×. The cell cytoskeleton was stained for 45 min with tetramethylrhodamine (TRITC, Sigma-Aldrich) at a 1:200 ratio in PBS 1×, followed by their nucleus staining for 5 min, with 4′,6-diamidino-2-phenylindole (DAPI, Sigma-Aldrich) at a 1:1000 ratio in PBS 1×. The staining method was carried out in the dark and at room temperature. An Olympus BX51 fluorescence microscope was used to visualize the samples.

## 3 Results and Discussion

### 3.1 Pristine PHBV Films

#### 3.1.1 Sample Morphology and the Degree of Porosity

**Figure 1:**
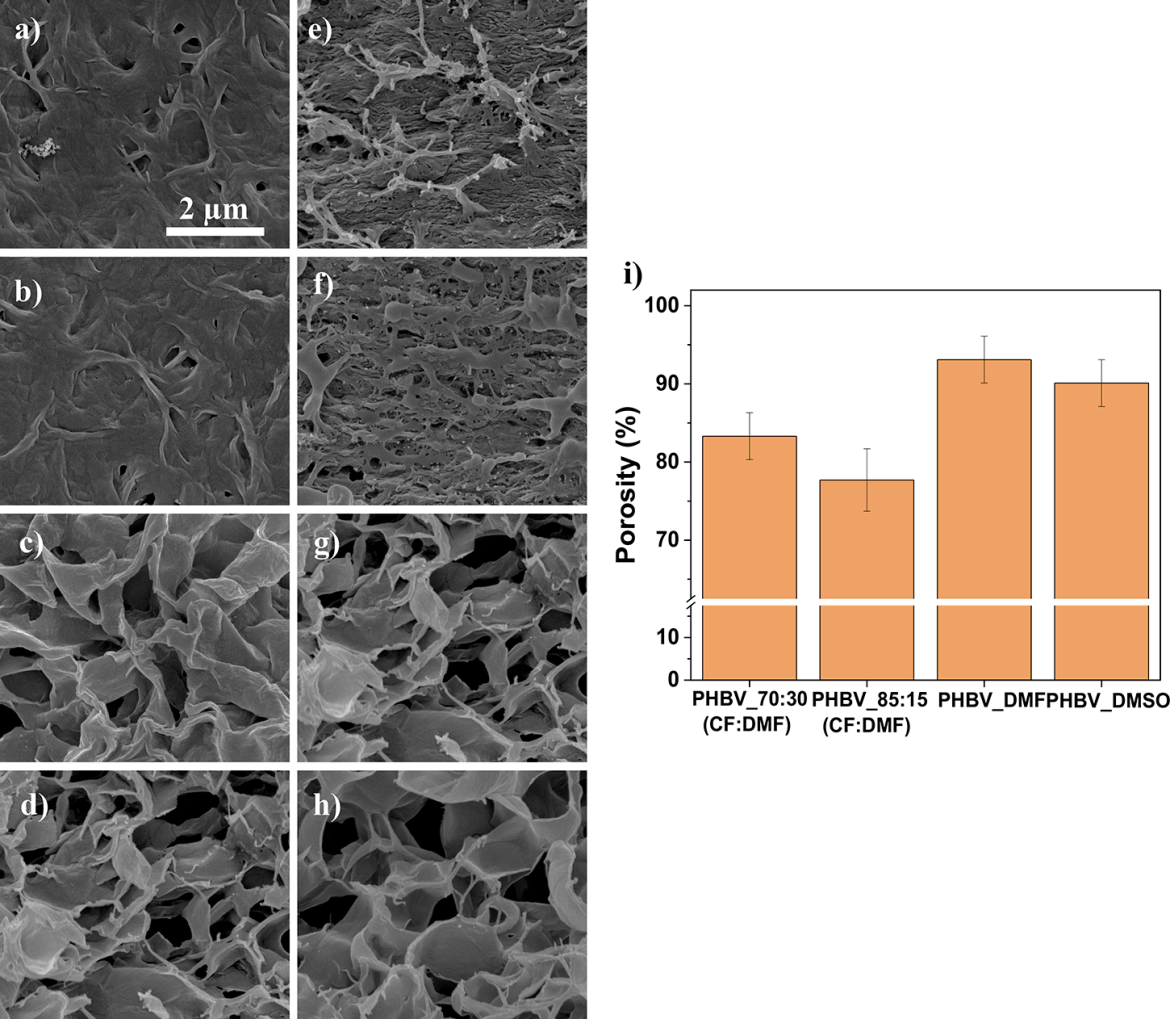
Surface and cross-section SEM micrographs of the different PHBV films surfaces of (a) PHBV_70:30 (CF:DMF), (b) PHBV_85:15 (CF:DMF), (c) PHBV_DMF, (d) PHBV_DMSO; and cross-sections of (e) PHBV_70:30 (CF:DMF), (f) PHBV_85:15 (CF:DMF), (g) PHBV_DMF, (h) PHBV_DMSO. Scale with 2 μm is representative for all images. (i) Degree of porosity of the different PHBV films.

The morphological features of the obtained films are observed in the representative SEM images presented in Figure 1.

Films developed based on the binary solvent system of CF and DMF present a surface with small pores in the range 0.1 to 0.7 μm (Figure 1a,b), while PHBV dissolved in DMF and DMSO is characterized by larger pores in the range 0.4 to 11 μm (Figure 1c, d). Films processed through the binary solvent systems of 70:30 and 85:15 (v/v) present similar thickness, 29 and 32 μm, respectively (Figure 1e,f), whereas films prepared after dissolving in DMF and DMSO present an average thickness of 105 and 74 μm, respectively (Figure 1g,h). The considerable variation on thickness between the binary systems and unitary systems is related to size variation of the obtained porous, larger pores leading to more void spaces and films with larger thicknesses, as confirmed by the degree of porosity of the films (Figure 1i).

Regarding the films produced through the binary solvent system, the degree of porosity is larger when larger DMF content is used, being with 83% for the 70:30 (CF:DMF) solution and 78% for the 85:15 (CF:DMF) ones. Films obtained from DMF and DMSO solutions show a degree of porosity around 90%. The use of solvents with low boiling temperature favors fast drying^36^ and the use of solvents with high boiling temperature provides enough time for the crystallization process, enabling a highly crystalline growth.^37^ In this way, the exclusive use of high-boiling-point solvents, such as DMF and DMSO, allows to obtain films with large degrees of porosity based on the fact that lower boiling temperature leads to higher volatility and shorter phase-separation time during the process, resulting in a reduction of the film porosity.^38^ The introduction of the binary system allows modulating the degree of porosity and pore size, in order to adapt the morphology to the specific needs of the application.

#### 3.1.2 Chemical, Thermal, and Wettability Characteristics

To analyze eventual physical–chemical modifications that took place based on the different processing conditions, FTIR-ATR and DSC measurements were performed, together with surface contact angle measurements to evaluate sample wettability.

**Figure 2:**
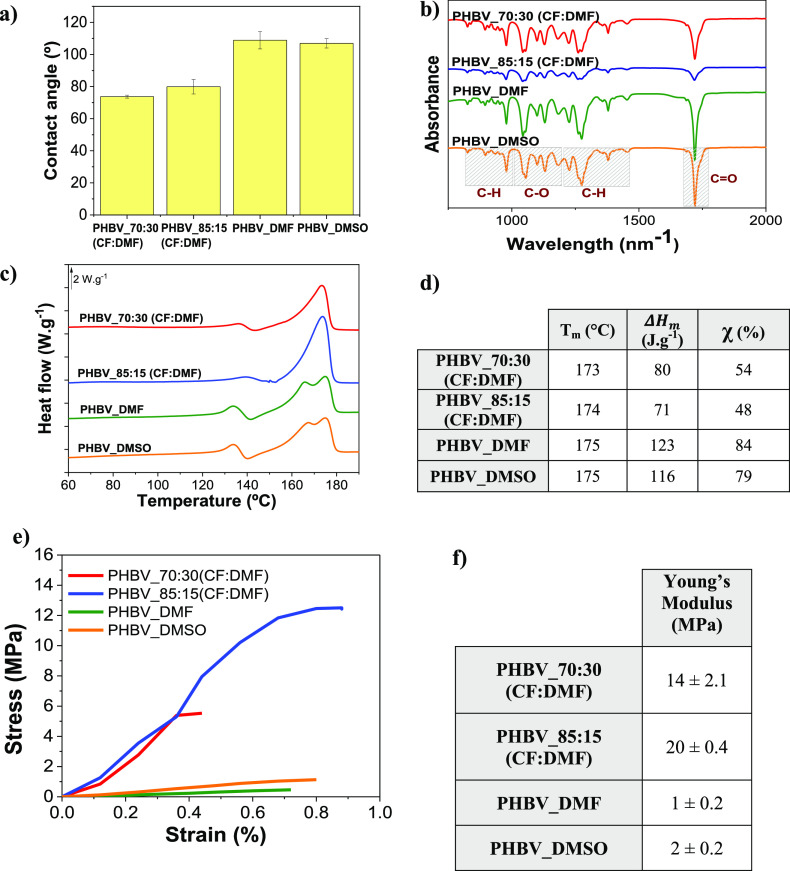
**(**a) Contact angle measurements of the PHBV films, (b) FTIR-ATR spectra, (c) DSC thermogram, and (d) respective *T*_m_, Δ*H*_m_, and χ (the associated error is ±2%). Representation of (e) mechanical stress–strain curves of the PHBV films and (f) corresponding Young’s modulus.

Contact angle evaluation is important for different applications because it provides material wettability, providing information on the behavior of the material in contact with liquids. For example, in vitro studies in biomedical applications are typically required for hydrophilic membranes to improve cell attachment.^39^ High contact angles characterize hydrophobic surfaces (>90°), while low contact angles are an indication of hydrophilic surfaces (<90°). Regarding contact angles measurements, Figure 2a, binary solvent systems lead to a hydrophilic behavior, with contact angles of 74° and 80° for the 70:30 (CF:DMF) and 85:15 (CF:DMF), respectively. On the other hand, PHBV dissolved in DMF or DMSO presented a hydrophobic behavior, with contact angles of 109° and 107°, respectively; the results are related to the differences in porosity and surface rugosity, and samples with higher porosity show a more hydrophobic behavior.^40^

The FTIR-ATR spectra (Figure 2b) allow to conclude that the processing does not induce any modification of the polymer, and all samples show the characteristic absorption bands of PHBV. Thus, bands at 820–980 and 1220–1460 cm^–1^ are observed, characterizing aliphatic C–H vibrational modes; at 1055, 1129, and 1180 cm^–1^, related to C–O vibrations, and at 1720 cm^–1^, characteristic of C=O stretching.^16^

Regarding DSC (Figure 2c), the melting peak of PHBV between 160 and 180 °C is observed for all the samples, as well as a small shoulder at the ascending branch of the main peak, around 130 °C. This shoulder has been reported in PHBV and has been ascribed to different crystalline phases with different sizes, thickness, and/or ordering.^41^ In particular, small and less perfect crystals melt at lower temperatures.^42^ Regarding PHBV dissolved with DMF and DMSO, the melting peak seems to be divided in two main events, one around 165 °C and the other around 175 °C, showing the formation of ill-crystallized or defective crystalline structures at the interfaces of the well-crystallized areas.^41^ Enthalpy (Δ*H*_m_) and crystalline percentages (χ) were estimated after eq 3, where Δ*H*_m_ represents the experimental melting enthalpy (J g^–1^) and Δ*H*_m_^0^ represents the theoretical enthalpy value of 100% crystalline PHBV (146.6 J g^–1^).^16^
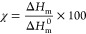
3

The use of high-boiling-point solvents leads to an increase of the degree of crystallinity of the samples, in particular for the samples processed with DMF and DMSO solvents (Figure 2d). For the binary solvent systems, the crystallization process occurs faster, leading to the formation of smaller crystallites and lower degrees of crystallinity. On the other hand, the use of high-boiling-point solvents leads to a slower crystallization process, larger spherulites, and larger degrees of crystallinity.^43^

#### 3.1.3 Mechanical Characterization

The mechanical response of the processed films was evaluated by tensile stress–strain tests. Mechanical measurements are presented in Figure 2e and the corresponding Young’s modulus in Figure 2f.

The mechanical response of the samples prepared from the binary solvent systems is characterized by an elastic region up to 0.3% strain, followed by a plastic region until rupture at 0.44% for 70:30 (CF:DMF) and 0.88% for 85:15 (CF:DMF). Films processed from DMF and DMSO exhibit an elastic behavior with a linear response until final rupture at 0.72 and 0.80% for DMSO and DMF, respectively. All curves are indicative of brittle materials.

PHBV films obtained through binary solvent systems present a higher mechanical stiffness compared to the dissolution in DMF and DMSO, with Young’s modulus of 14 MPa for 70:30 (CF:DMF) and 20 MPa for 85:15 (CF:DMF) films. PHBV with DMF presents a1 MPa Young’s modulus and with DMSO 2MPa. Pores act as defects; thus, mechanical properties are highly influenced by material porosity. With higher porosities, tensile strength and Young’s modulus tend to decrease, diminishing mechanical performance.^44,45^ Besides the effect of morphology, mechanical properties are also strongly related to the crystallization process, the maximum strain decreases with increasing spherulite average size.^43^ As obtained from the DSC analysis, PHBV_DMF and PHBV_DMSO films present higher crystallinity degrees, 84 and 79%, respectively, which can be related to larger spherulites causing poor mechanical properties.

#### 3.1.4 Topography and Local Piezoelectric Response

PHBV is a piezoelectric polymer,^46^ although its piezoelectric response has been scarcely addressed.

**Figure 3:**
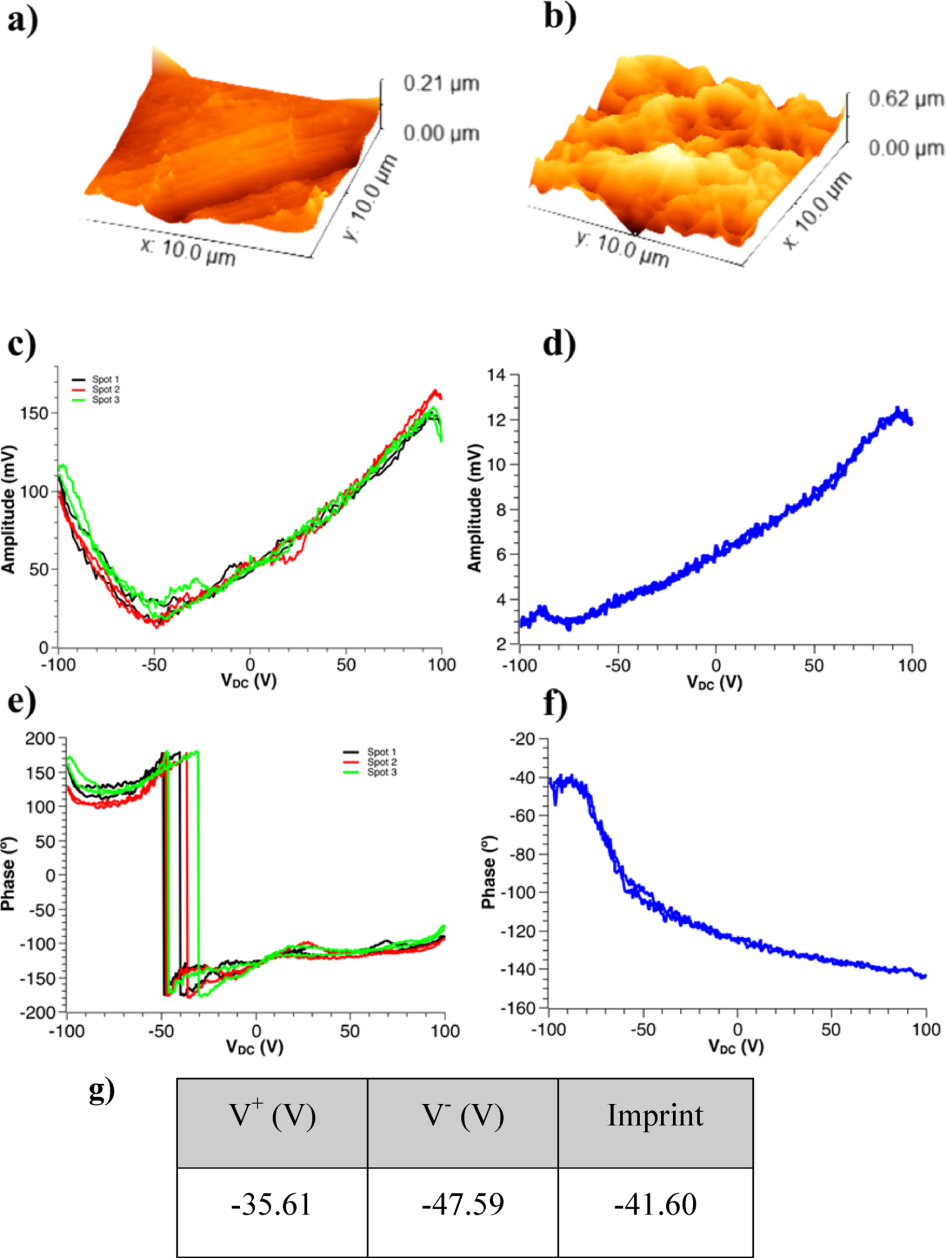
3D topography of the (a) poled and (b) nonpoled PHBV dense films; amplitude of the piezoelectric response of the (c) poled and (d) nonpoled PHBV films; phase shift in the (e) poled and (f) nonpoled PHBV films; and (g) mean of measurements of the forward bias, reverse bias, and imprint of the hysteresis loops of the PHBV poled film.

Figure 3 shows the 3D topography, response amplitude, and phase shift of the poled and nonpoled PHBV using the CF solvent (dense samples).

The roughness of the surface was evaluated as the arithmetic roughness (*R*_a_) (eq 4) and the root-mean-squared roughness (*R*_q_) (eq 5):^47^
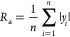
4
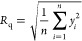
5where *y_i_* indicates each of the individual points of the height image. After eliminating the possible outliers in the image and performing a first-order polynomial leveling on the images, a *R*_a_ value of 74.51 nm for the nonpoled sample and 13.76 nm for the poled sample was obtained. Similarly, the *R*_q_ values were 92.95 and 17.18 nm, respectively. This is clearly observed in the 3D topography images, where the poled sample (Figure 3a) shows significantly lower variation in height than the nonpoled one (Figure 3b), attributed to the effect of the high-energy corona discharge process.

After obtaining the images, the local piezoelectric response was evaluated by applying a DC bias through the cantilever. The application of this voltage generates an electric field of several kilovolts per centimeter, higher than the coercive voltage of most ferroelectric materials, thus inducing local polarization reversal.^32^ Then, this switching is investigated by sweeping this DC bias while scanning the sample. In the phase image, the voltage at which the reversal of the polarization occurs can be observed. On the other hand, the amplitude curve gives an idea of the strength of that piezoelectric response. In the case of the nonpoled sample (Figure 3d,f), no sign of ferroelectric switching is observed, as indicated by the absence of the hysteresis loop of the phase. On the contrary, the poled sample (Figure 3c,e) shows polarization reversal, as measured in different zones of the sample. This switching was captured at three different spots of the sample to give three different values of the forward (*V^+^*) and reverse (*V^–^*) coercive biases, as well as the imprint of the loop, which defines the asymmetry of the hysteresis, defined by eq 6, and the results are presented in Figure 3g.
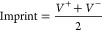
6

Figure 3c,e shows that the minimum of the amplitude loops coincides with the point at which the phase suffers a shift of 180°, which indicates the presence of two stable states with opposite polarity.^48^ It was also observed that the phase loops are significantly shifted toward the negative voltage values (Figure 3g), which can be due to an imprint phenomenon in the local switching properties,^49,50^ related to the presence of an internal built-in electric field which supports one polarization state while opposing the antiparallel.^34^

### 3.2 Composite PHBV/Fe_3_O_4_ Films

#### 3.2.1 Morphological Evaluation

**Figure 4:**
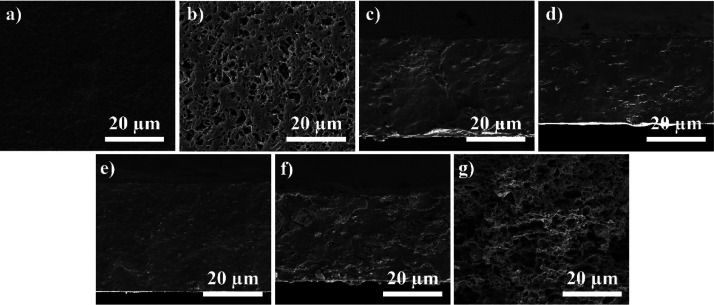
Surfaces micrographs of **(**a) representative neat and dense PHBV surface and (b) porous 10% Fe_3_O_4_ (CF/DMF); and cross-sectional micrographs of neat and composite films: (c) neat PHBV, (d) PHBV + 5% Fe_3_O_4_, (e) PHBV + 10% Fe_3_O_4_, (f) PHBV + 20% Fe_3_O_4_, and (g) PHBV + 10% Fe_3_O_4_ (CF/DMF).

Neat PHBV and composite PHBV films with 5, 10, and 20% of Fe_3_O_4_ contents were processed, under dense morphology (without pores). Furthermore, the use of the binary solvent system (CF and DMF) in a 70:30 v/v concentration was used to process porous composite films with 10% Fe_3_O_4_ content. The morphology of the PHBV films was evaluated by SEM, as presented in Figure 4.

All dense PHBV films (neat and composite) reveal a similar surface micrograph; thus, just a representative image corresponding to pristine PHVB is provided in Figure 4a. The surface of the films is characterized by a compact microstructure, independent of the filler content, indicating that nanoparticles are in the bulk of the PHBV matrix. Figure 4b shows a porous surface micrograph, with pores ranging from 0.3 to 5 μm and 90% porosity, measured by the liquid displacement method, proving the successful processing of porous composite films of the binary solvent system.

Cross-section images of the films also allow to identify the presence of the nanoparticles all along the thickness of the composite films. The porous composite film is characterized by a homogeneous distribution of interconnected pores all along the thickness of the sample. The average thickness of the samples is 31 μm for PHBV neat films, 28 μm for PHBV with 5% Fe_3_O_4_, 36 μm for with 10% Fe_3_O_4_, 25 μm for 20% Fe_3_O_4_ content, and 56 μm for the PHBV with 10% Fe_3_O_4_ developed under a binary solvent system of CF and DMF. The last one presents a considerably higher thickness because of the porous microstructure. EDS analysis (see Table S1 of Supporting Information) allows to confirm the weight percentage of iron (Fe) in the PHBV composite, which agrees with the nanoparticle content added during the development of the films, demonstrating good efficiency of the method.

#### 3.2.2 Physical–Chemical and Wettability Characterization

To disclose any physical–chemical modifications during the inclusion of the Fe_3_O_4_ nanoparticles, FTIR-ATR, DSC, and TGA measurements were performed, as well as contact angle measurements to assess film wettability.

**Figure 5:**
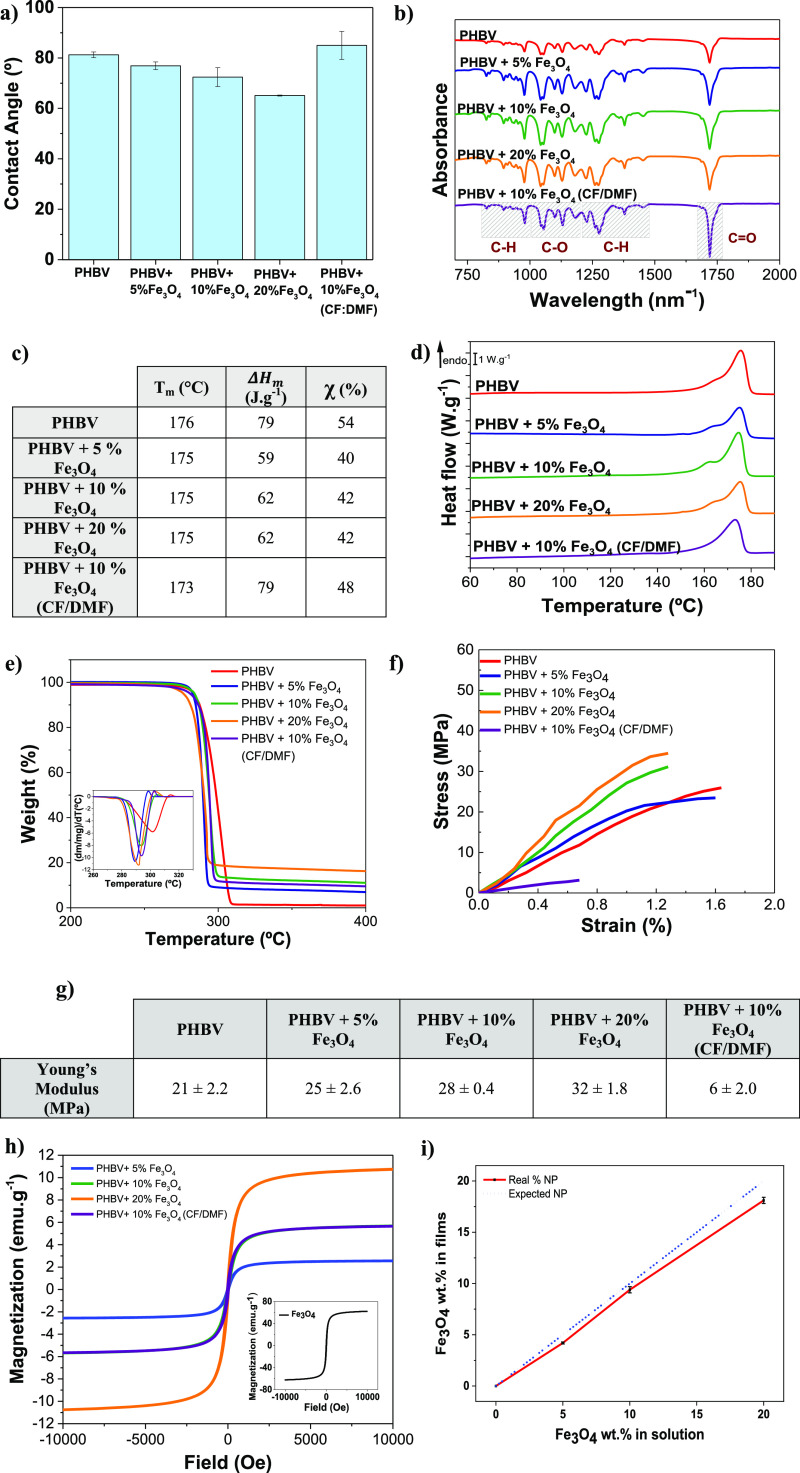
Representative (a) contact angle measurements of neat and PHBV composite films, (b) FTIR spectra, (c) DSC thermogram, respective (d) *T*_m_, Δ*H*_m_, and χ, and (e) TGA thermogram with corresponding first derivatives; (f) mechanical tensile stress–strain response of neat and composite PHBV films and (g) corresponding Young’s modulus; **(**h) room temperature hysteresis curves for the Fe_3_O_4_/PHBV composites with pure Fe_3_O_4_ hysteresis curve inset and (i) real nanoparticle content and respective inclusion efficiency.

Regarding contact angle measurements presented in Figure 5a, surface wettability diminishes with the inclusion of nanoparticles and with increasing filler content, as reported in the literature.^51^ Neat PHBV presents an 81° contact angle, while composite films present lower values: 77° for 5%, 72° for 10%, and 65° for 20% of Fe_3_O_4_ content. The increasing content of Fe_3_O_4_ nanoparticles leads to a decrease of the contact angle and consequently an increase of film hydrophilicity, which is explained by the good hydrophilicity of Fe_3_O_4._^52^ Regarding composite porous films, they exhibit the highest contact angle (85°), which is attributed to their porous morphology that leads to a higher surface roughness, compared to dense substrates.^53^

Figure 5b presents FTIR-ATR spectra of all the films developed, including neat PHBV and Fe_3_O_4_/PHBV with 5, 10, and 20% contents of Fe_3_O_4_, as well as porous PHBV with 10% Fe_3_O_4_ produced with the binary solvent system. No differences are observed in the absorption bands of PHBV after the inclusion of Fe_3_O_4_. As occurred for porous films, characteristic PHBV absorption bands are detected, such as in the region of 820–980 and 1220–1460 cm^–1^ that characterize aliphatic C–H vibrational modes. C–O vibrations can be at around 1055, 1129, and 1180 cm^–1^ and a C=O stretching at 1720 cm^–1^. These results indicates that no structural modification (chemical bonding) took place with the insertion of the nanoparticles, but just electrostatic interaction between the filler and the polymer.^16^

DSC thermograms (Figure 5d) show a small shoulder for 5, 10, and 20% Fe_3_O_4_ contents, at the ascending branch of the main peak (around 165 °C) which indicates that the presence of the nanofillers leads to variations in the crystallization process, leading to more defective crystalline structures.^41,54^ Around 175 °C, the PHBV melting peak is observed in all samples, as represented in Figure 5c together with Δ*H*_m_ and the degree of crystallinity (χ). The inclusion of magnetic nanoparticles leads to a slight decrease in the degree of crystallinity, and the filler creates defects that hinder spherulite growth.^16^

TGA curves of neat and composite PHBV films are shown in Figure 5e, which demonstrates a one-step thermal degradation for all samples. PHBV thermally degrades by a random one-step β-elimination chain scission reaction, around 303 °C.^55^ With the inclusion of the magnetic nanoparticles, a slight decrease in the maximum temperature before degradation, around 290 °C (for 5 and 10% Fe_3_O_4_) and 292 °C (for 20 and 10% (CF/DMF)), it is better identified with the first derivatives inset graph. This early thermal degradation, and consequent decrease in thermal stability in the composite films, is explained by the larger thermal conductivity of Fe_3_O_4_ with respect to the polymer matrix.^16^ It is also observed that neat films degrade with negligible residues, whereas PHBV/Fe_3_O_4_ films of 5% of nanoparticles leave about 7% residue, 10% Fe_3_O_4_ porous and nonporous leaves about 9.5 and 11%, respectively, and 20% Fe_3_O_4_ leaves around 16% of residue. These residues correlate with the content of nanoparticles.

#### 3.2.3 Mechanic and Magnetic Characterization

Stress–strain tests were performed to evaluate the mechanical response of all processed films. The mechanical measurements are presented in Figure 5f and the corresponding Young’s modulus in Figure 5g.

All films exhibit a brittle behavior, with the linear elastic region along approximately 1% of strain, followed by a short plastic region until rupture, around 1.7% for neat PHBV and PHBV + 5% Fe_3_O_4_ and around 1.3% of strain for PHBV films with 10 and 20% nanoparticle contents. Porous composite PHBV films produced with the binary solvent system present a linear elastic region until 0.5% and a short plastic region until 0.7% when it breaks, also showing a brittle behavior. This demonstrates the strong effect of the processing method and sample morphology on the material’s mechanical characteristics. The incorporation of nanoparticles diminishes the ultimate tensile strength of the films, but increases Young’s modulus, as it has been reported before, because of the electrostatic interaction that occurs between the filler and the polymer.^16^ For the porous composite film, Young’s modulus was significantly lower, which is explained by its porous morphology, as previously discussed.

Magnetic properties of the Fe_3_O_4_/PHBV films were assessed by VSM, as well as the quantification of the nanoparticles content, with magnetization curves being presented in Figure 5h,i.

The hysteresis curves reveal a typical superparamagnetic behavior for the nanocomposites with Fe_3_O_4_ nanoparticles and increasing magnetization with the increase of the magnetic field until saturation is reached (Figure 5h). Pure Fe_3_O_4_ nanoparticles reveal a saturation magnetization of 62 emu g^–1^ observed at 10 kOe, while composite films present considerably lower maximum magnetizations, decreasing with decreasing nanoparticle content, being about 10, 6, and 3 emu g^–1^ for 20, 10, and 5% Fe_3_O_4_ contents, respectively.

VSM is a precise technique that enables to obtain the real Fe_3_O_4_ content after film processing, resorting to eq 7, where Ms_sample_ is the saturation magnetization measured in the sample and Ms_0_ the saturation magnetization measured in pure Fe_3_O_4_.^56^
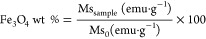
7

Figure 5i shows the effective nanoparticle content in the different PHBV composites versus the theoretical values. The results demonstrated that the nanoparticle content incorporated in the composites films is close to the theoretical value, showing good efficiency of the process. Thus, the results demonstrate high efficiencies in the nanoparticle incorporation for all samples (from 84 to 90%), agreeing with EDS analysis previously presented. The small difference between the expected and experimental magnetic saturation values is explained by the settling of some nanoparticles on the nanocomposites’ mixing container, resulting from their higher density when compared to the polymer matrix.

### 3.3 Material Evaluation for Biomedical Applications

#### 3.3.1 Magnetic and Mechanical Stimulus of the Composite Films

In the biomedical context, many approaches are being used in an attempt to promote cell proliferation and differentiation by a biomimetic recreation of specific cell microenvironments. Advanced materials with active features are being increasingly investigated in association with new generation bioreactors.^57^ Chemical, electrical, magnetic, mechanic, and optical stimuli can be used at different levels, according to the cell type and final purpose.^57^

The developed composite materials can be used as magnetomechanical and magnetoelectrical scaffolds for in vitro studies, taking advantage of the application of a variable magnetic field that can be produced by a magnetic bioreactor.^15^ The magnetoelectric response is ascribed to the magnetostrictive phase (Fe_3_O_4_) and the piezoelectric phase (PHBV), whose coupling is mediated by the strain. When the scaffold is submitted to a varying magnetic field, the Fe_3_O_4_ nanoparticles will undergo volumetric size variations (magnetostrictive effect), as well as small displacements from the equilibrium positions, that is transduced to the PHBV that, in turn, will originate an electrical response as a response to the mechanical solicitation (piezoelectric effect).^58^

After the magnetic field variation, exerted by the bioreactor, there will be an associated scaffold mechanical response which is important to be characterized. The mechanical stimulus sensed by the cells is the one triggering the electrical response in a piezoelectric sample.^15^ Considering that the stimulus has a dynamic behavior, the response is also expected to follow a similar dynamic. Thus, to better understand the various phenomena, an experimental setup was developed to measure the strain that would be effectively applied to the cell surface when in contact with the 5% Fe_3_O_4_/PHBV, 10% Fe_3_O_4_/PHBV, and 20% Fe_3_O_4_/PHBV composite films. Films were introduced into a 24-well culture plate with cell culture medium, placed in a custom-made magnetic bioreactor,^15^ and subjected to a variable magnetic field. In the biological tests, a linear displacement of 19 mm was applied at a frequency of 0.6 Hz. The evaluation of the results of the magnetic fields in the center of the well and the variation of the strain gauge were also performed in the same conditions (approximately 40 cycles per minute).

**Figure 6:**
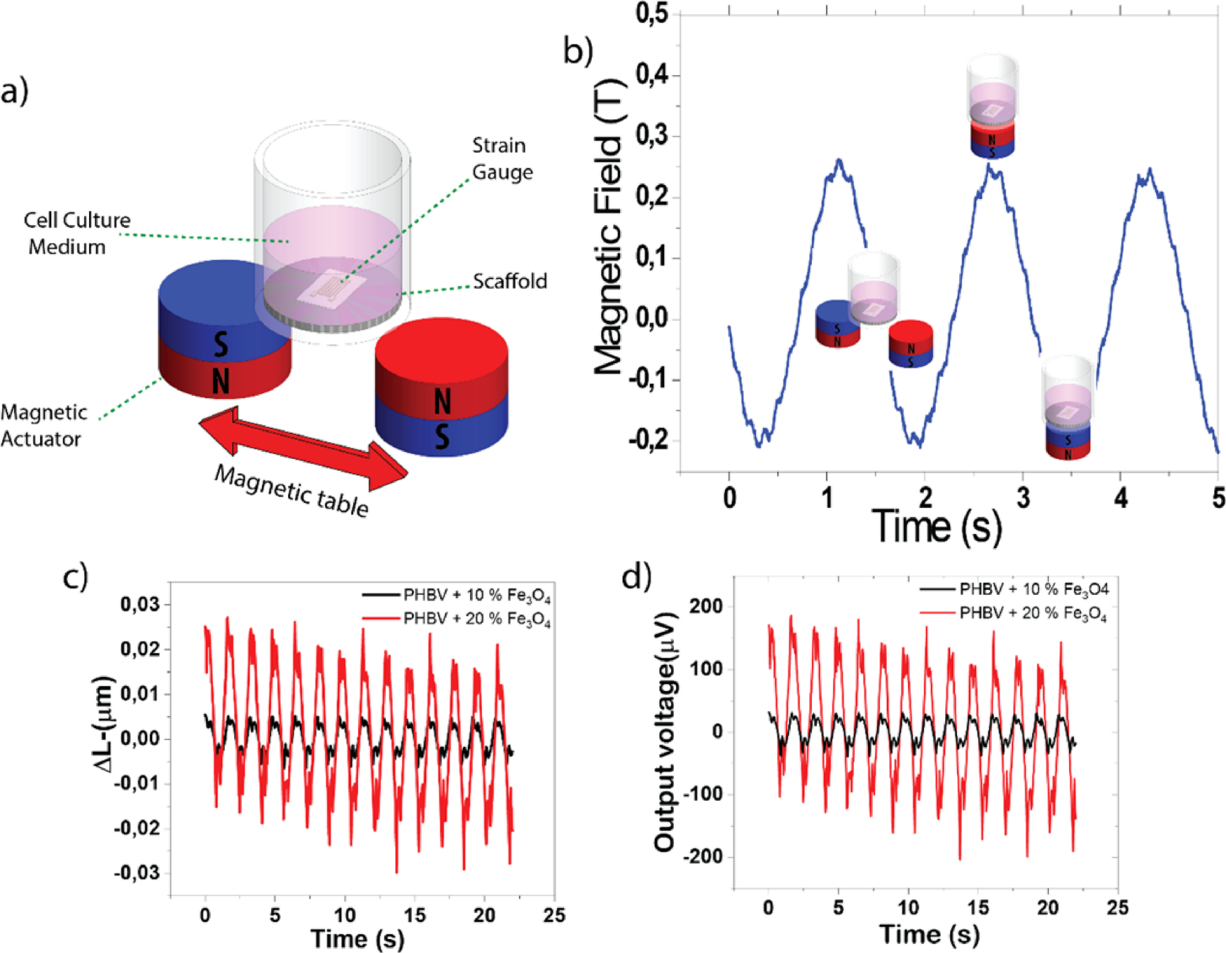
(a) Schematic representation of the magnetic stimulus operation method, with the identification of the various constituent parts. (b) Magnetic field measurement to which the scaffold is subjected, according to its relative position with respect to the magnetic table. (c) Deformation measurement, obtained from the measured sensor resistance variation for different scaffold materials. (d) Calculated voltage generated by the scaffold in response to the mechanical variation produced by the magnetic variation.

Given the characteristics of the material used, the first major transduction that occurs corresponds to a magnetic stimulus that translates into an applied mechanical stimulus. First of all, the maximum and minimum magnetic field perpendicular to the film surface to which the scaffold is subjected was measured using a gaussmeter, showing that the magnetic field to which the scaffold is subjected varies between 0.22 and −0.22 T, given the position of the magnets in relation to the scaffold. The monitoring of magnetic field over the time according to the relative position between the magnet table and the center of the cell culture well was achieved using a Linear Output Magnetic Field Sensor AD22151 from Analog Devices that presents an internal sensitivity of 0.4 mV G^–1^ (for a gain of 1), connected to a 16-bit high resolution digital oscilloscope (PicoScope 5242D). The voltage response as a function of time results in the field variation according to Figure 6b.

Figure 6a,b shows the variation of the produced magnetic field, being approximately zero in the central regions between the magnets. By analyzing the external regions of the magnetic table, a slightly higher magnetic field (0.23 to −0.23 T) was verified in the periphery of the cell culture well when compared to the central region because of the lack of another set of magnets that allows the uniformity of the field.

The measurement of the mechanical deformation of the different scaffolds was carried out (Figure 6c). For this purpose, a strain gauge sensor was coupled to the scaffold, in which the resistance variation was measured (digital multimeter Rigol DM3068) under bioreactor operation (Figure 6c). It is important to note that the sensor was placed at the center of the cultivation well, concentric with the bioreactor excitation magnet, in which a displacement cycle was applied that allowed the table to be moved until the sensor was concentric with the neighbor excitation, corresponding to a total displacement of 19 mm, with a frequency of 0.6 Hz. In this way, a maximum magnetic excitation of approximately 0.22 T (Figure 6b) is applied.

Because the objective is to obtain the mechanical variation of the sample, the conversion of the resistance to the deformation variation is carried out, according to the gauge factor equation (eq 8), where the gauge factor of the sensor is 2.105 +/– 0.5% and its length along the axis of measurement is *L*_0_ = 1.52 mm, with *R*_0_ being the resistance of the undeformed gauge.
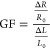
8

Thus, after this conversion, it was possible to obtain the dimensional variation of the sample over the various magnetic excitation cycles, as shown by Figure 6c, for the different materials.

By analyzing the response graph, it is verified that the materials follow the stimulus format represented in Figure 6c, keeping their response relatively stable over time. By comparing the various materials, it is verified that the PHBV films with 20% Fe_3_O_4_ present the highest mechanical variation, corresponding to a dimension variation of approximately 0.036 μm, while the PHBV with 10% Fe_3_O_4_ shows a size range of approximately 0.011 μm. These variations correspond to a variation per unit area of 0.024 μm mm^–2^ for samples with 20% Fe_3_O_4_ and 0.0073 μm mm^–2^ with 10% Fe_3_O_4_. The response of 5% Fe_3_O_4_/PHBV films was not possible to be measured because it was out the resolution range of the equipment, although it is expected that it would follow the same behavior but with a lower dimension variation. The mechanical response of the scaffolds is strongly conditioned by the magnetic stimuli as well as by its variation, thus, after these studies, it is possible to define adequate mechanical stimuli for the characteristics of the cells, by controlling the magnets distance and the Fe_3_O_4_ concentration.

Taking into account the piezoelectric characteristics of the base material, PHBV,^16^ it is possible to estimate of the output voltage (Figure 6d) generated by the scaffolds according to the equation that relates the output voltage to the piezoelectric characteristics (eq 9),
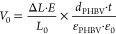
9where Δ*L* is the dimension variation, *E* is Young’s modulus, *L*_0_ is the initial length, *d* is the piezoelectric coefficient, *t* is the film thickness, ε is the relative dielectric permittivity, and ε_0_ is the permittivity of free space_._

Observing the output voltage response graph (Figure 6d), and comparing the two materials, it is verified that the PHBV films with 20% Fe_3_O_4_ present voltage peak-to-peak variations of approximately 250 μV, while the PHBV with 10% Fe_3_O_4_ has a voltage variation of approximately 50 μV. These dimensional variations correspond to a potential difference per unit area of 108 μV mm^–2^ for 20% Fe_3_O_4_ samples and 21 μV mm^–2^ for 10% Fe_3_O_4_ samples.

#### 3.3.2 Cytotoxicity Assay

**Figure 7:**
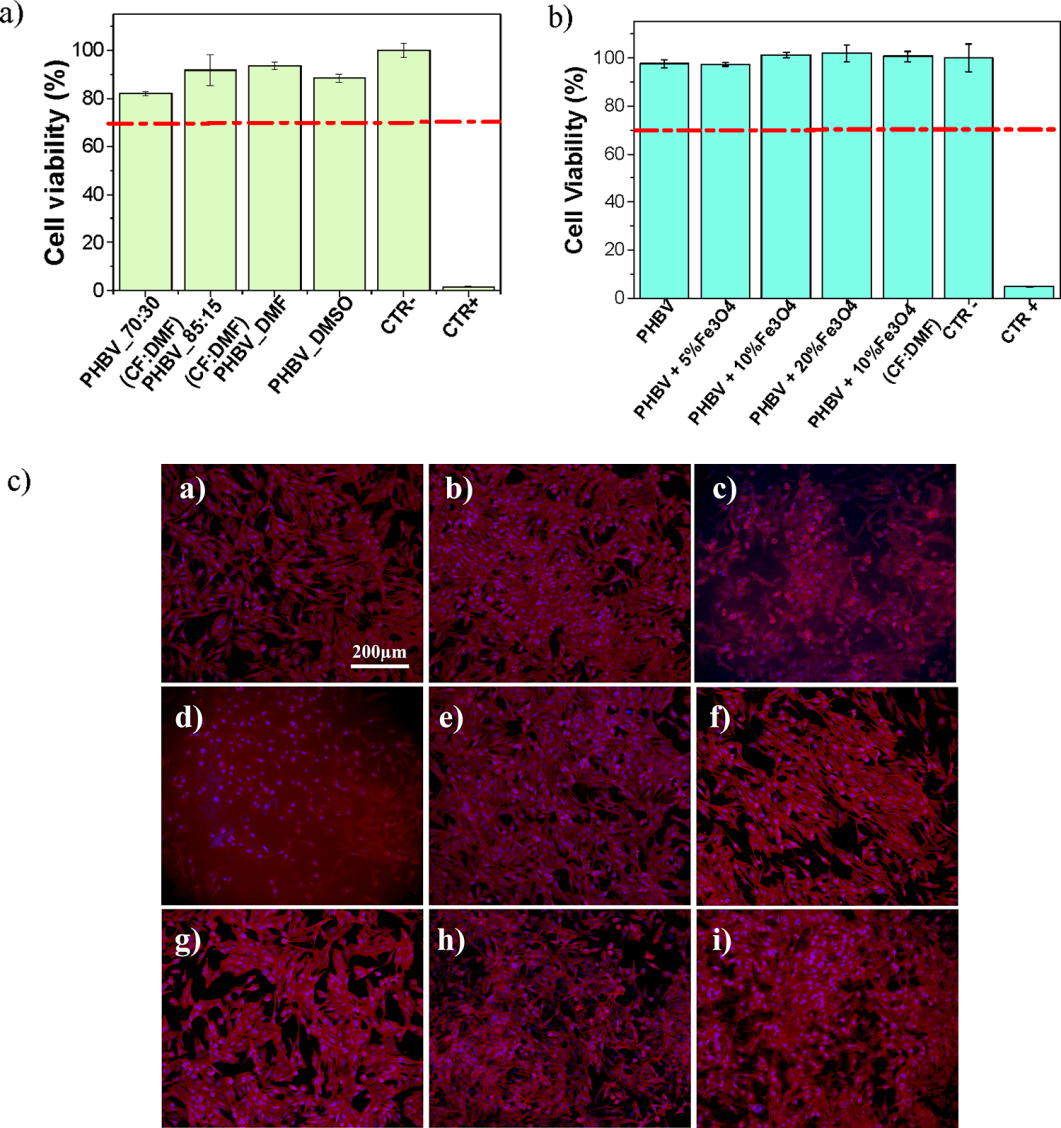
Cell viability evaluation of (a) PHBV porous films and (b) Fe_3_O_4_/PHBV composite films. Red line represents 70% threshold for cellular cytotoxicity and (c) immunofluorescence imaging of osteoblast culture on (i) PHBV_70:30 (CF:DMF), (ii) PHBV_85:15 (CF:DMF), (iii) PHBV_DMF, (iv) PHBV_DMSO, (v) PHBV, (vi) PHBV + 5% Fe_3_O_4_, (vii) PHBV + 10% Fe_3_O_4_, (viii) PHBV + 20% Fe_3_O_4_, and (ix) PHBV + 10 Fe_3_O_4_ (CF:DMF). Scale bar (200 μm) is valid for all the images.

To confirm the noncytotoxic behavior of the developed materials, indirect cytotoxicity tests were carried out. After 24 h of exposure to the materials, the respective media were placed in contact with osteoblast cells. After 72 h of contact, MTT assay was performed, and the results are shown in Figure 7.

It is verified that porous PHBV films do not induce cytotoxicity, with cell viabilities higher than 70% for all materials. Similarly, neat and composite Fe_3_O_4_/PHBV films are also noncytotoxic, with the metabolic activity superior to 97% for all the developed samples. These results agree with the literature, where both PHBV and Fe_3_O_4_ are identified as biocompatible.^16,31^

Following a 24 h culture period, cell adhesion of MC3T3-E1 cells was studied by observing cell cytoskeleton and nuclei morphology of adhered cells under immunofluorescence imaging (Figure 7c).

The results show clean images for almost all samples (Figure 7c: i–iii, v–ix), except for porous PHBV_DMSO (Figure 7c: iv) that present a higher background, because of the dispersion of fluorescent light, making the analysis more challenging. Moreover, in higher porosity samples as PHBV_DMF (Figure 7c: iii) and PHBV_10% Fe_3_O_4_(CF:DMF) (Figure 7c: ix), cells seem to penetrate the pores, which leads to the appearance of smaller cytoskeleton sizes hindering cell elongation and orientation.

In general, preosteoblast cells exhibit a similar number of nuclei in all substrates and consequently similar cell density, supporting the concept that these materials have the potential to be used in biomedical applications.

## 4 Conclusions

Porous PHBV films were successfully obtained by solvent casting, making use of high boiling temperature solvents, DMF and DMSO, as well as binary solvent systems of CF–DMF. Films developed using DMF and DMSO allow samples with a high degree of porosity with interconnected pores. PHBV films obtained through binary solvent systems present larger elastic modulus—14 MPa for PHBV_70:30 (CF:DMF) and 20 MPa for PHBV_85:15 (CF:DMF) – than the ones obtained with DMF and DMSO, 1 and 2 MPa, respectively. The piezoelectric response of PHBV was confirmed by PFM.

Moreover, up to 20% (w/w) Fe_3_O_4_ nanoparticles were incorporated into the PHBV films, showing good nanoparticle dispersion and incorporation efficiency. The nanoparticles act as mechanical reinforcement of the PHBV polymer, increasing the elasticity modulus of the PHBV + 20% Fe_3_O_4_ composite reaching Young’s modulus of 32 MPa. The saturation magnetization increases with filler content up to a value of 10 emu g^–1^ for the PHBV with a 20% concentration of Fe_3_O_4_. This allows a magnetomechanical stimulation up to 0.024 μm mm^–2^ for samples with 20% Fe_3_O_4_, which is accompanied by a magnetoelectric effect in the piezoelectric samples. Cell adhesion was evaluated in all the produced samples, and, in general, preosteoblasts cells exhibit a similar number of nuclei in all substrates. Thus, different types of porous and nonporous biocompatible films with piezoelectric and/or magnetoactive behavior have been developed as a platform for biotechnological applications.

## Supporting Information Available

Weight percentage of Fe in the different composite films obtained through EDS analysis (PDF)

## Notes

The data generated during the current study are available from the corresponding author upon reasonable request.

## References

[ref1] Joyce K.; Fabra G. T.; Bozkurt Y.; Pandit A. Bioactive potential of natural biomaterials: identification, retention and assessment of biological properties. Signal Transduct. Target. Ther. 2021, 6, 122 10.1038/s41392-021-00512-8. 33737507 PMC7973744

[ref2] Liao S.; Chan C. K.; Ramakrishna S. Stem cells and biomimetic materials strategies for tissue engineering. Mater. Sci. Eng., C 2008, 28, 1189–1202. 10.1016/j.msec.2008.08.015.

[ref3] Meira R. M.; Correia D. M.; Ribeiro S.; Costa P.; Gomes A. C.; Gama F. M.; Lanceros-Méndez S.; Ribeiro C. Ionic-Liquid-Based Electroactive Polymer Composites for Muscle Tissue Engineering. ACS Appl. Polym. Mater. 2019, 1, 2649–2658. 10.1021/acsapm.9b00566.

[ref4] Xiao L.; Chen X.; Yang X.; Sun J.; Geng J. Recent Advances in Polymer-Based Photothermal Materials for Biological Applications. ACS Appl. Polym. Mater. 2020, 2, 4273–4288. 10.1021/acsapm.0c00711.

[ref5] Xin Y.; Liu T.; Sun H.; Xu Y.; Zhu J.; Qian C.; Lin T. Recent progress on the wearable devices based on piezoelectric sensors. Ferroelectrics 2018, 531, 102–113. 10.1080/00150193.2018.1497411.

[ref6] Zaszczyńska A.; Gradys A.; Sajkiewicz P. Progress in the applications of smart piezoelectric materials for medical devices. Polymer 2020, 12, 2754 10.3390/polym12112754. PMC7700596 33266424

[ref7] Aliqué M.; Simão C. D.; Murillo G.; Moya A. Fully-Printed Piezoelectric Devices for Flexible Electronics Applications. Adv. Mater. Technol. 2021, 6, 2001020 10.1002/admt.202001020.

[ref8] Ribeiro S.; Gomes A. C.; Etxebarria I.; Lanceros-Méndez S.; Ribeiro C. Electroactive biomaterial surface engineering effects on muscle cells differentiation. Mater. Sci. Eng., C 2018, 92, 868–874. 10.1016/j.msec.2018.07.044. 30184816

[ref9] Dalgic A. D.; Koman E.; Karatas A.; Tezcaner A.; Keskin D. Natural origin bilayer pullulan-PHBV scaffold for wound healing applications. Mater. Sci. Eng., C 2021, 6, 112554 10.1016/j.msec.2021.112554. 35523643

[ref10] Tandon B.; Magaz A.; Balint R.; Blaker J. J.; Cartmell S. H. Electroactive biomaterials: Vehicles for controlled delivery of therapeutic agents for drug delivery and tissue regeneration. Adv. Drug Delivery Rev. 2018, 129, 148–168. 10.1016/j.addr.2017.12.012. 29262296

[ref11] Xu L.; Yang Y.; Mao Y.; Li Z. Self-Powerbility in Electrical Stimulation Drug Delivery System. Adv. Mater. Technol. 2021, 7, 2100055 10.1002/admt.202100055.

[ref12] Ribeiro C.; Sencadas V.; Correia D. M.; Lanceros-Méndez S. Piezoelectric polymers as biomaterials for tissue engineering applications. Colloids Surf., B 2015, 136, 46–55. 10.1016/j.colsurfb.2015.08.043. 26355812

[ref13] Marino A.; Genchi G. G.; Sinibaldi E.; Ciofani G. Piezoelectric Effects of Materials on Bio-Interfaces. ACS Appl. Mater. Interfaces 2017, 9, 17663–17680. 10.1021/acsami.7b04323. 28485910

[ref14] Marino A.; Genchi G. G.; Mattoli V.; Ciofani G. Piezoelectric nanotransducers: The future of neural stimulation. Nano Today 2017, 14, 9–12. 10.1016/j.nantod.2016.12.005.

[ref15] Ribeiro S.; Ribeiro C.; Carvalho E. O.; Tubio C. R.; Castro N.; Pereira N.; Correia V.; Gomes A. C.; Lanceros-Méndez S. Magnetically Activated Electroactive Microenvironments for Skeletal Muscle Tissue Regeneration. ACS Appl. Bio Mater. 2020, 3, 4239–4252. 10.1021/acsabm.0c00315. 35025425

[ref916] Jacob J.; More N.; Kalia K.; Kapusetti G.; et al. Piezoelectric smart biomaterials for bone and cartilage tissue engineering. Inflammation and Regeneration 2018, 38, 2 10.1186/s41232-018-0059-8. 29497465 PMC5828134

[ref16] Amaro L.; Correia D.; Marques-Almeida T.; Martins P.; Pérez L.; Vilas J.; Botelho G.; Lanceros-Mendez S.; Ribeiro C. Tailored biodegradable and electroactive poly (hydroxybutyrate-co-hydroxyvalerate) based morphologies for tissue engineering applications. Int. J. Mol. Sci. 2018, 19, 2149 10.3390/ijms19082149. 30042300 PMC6121965

[ref17] Rivera-Briso A. L.; Serrano-Aroca Á. Poly(3-Hydroxybutyrate-co-3-Hydroxyvalerate): Enhancement strategies for advanced applications. Polymer 2018, 10, 732 10.3390/polym10070732. PMC6403723 30960657

[ref18] Goonoo N.; Gimié F.; Ait-Arsa I.; Cordonin C.; Andries J.; Jhurry D.; Bhaw-Luximon A. Piezoelectric core-shell PHBV/PDX blend scaffolds for reduced superficial wound contraction and scarless tissue regeneration. Biomater. Sci. 2021, 9, 5259–5274. 10.1039/D1BM00379H. 34164641

[ref19] Fernandes J. G.; Correia D. M.; Botelho G.; Padrão J.; Dourado F.; Ribeiro C.; Lanceros-Méndez S.; Sencadas V. PHB-PEO electrospun fiber membranes containing chlorhexidine for drug delivery applications. Polym. Test. 2014, 34, 64–71. 10.1016/j.polymertesting.2013.12.007.

[ref20] Carlos A. L. M.; Mancipe J. M. A.; Dias M. L.; Thiré R. M. S. M. Poly(3-hydroxybutyrate-co-3-hydroxyvalerate) core-shell spun fibers produced by solution blow spinning for bioactive agent’s encapsulation. J. Appl. Polym. Sci. 2022, 139, 52081 10.1002/app.52081.

[ref21] Chernozem R. V.; Pariy I. O.; Pryadko A.; Bonartsev A. P.; Voinova V. V.; Zhuikov V. A.; Makhina T. K.; Bonartseva G. A.; Shaitan K. V.; Shvartsman V. V.; Lupascu D. C.; Romanyuk K. N.; Kholkin A. L.; Surmenev R. A.; Surmeneva M. A. A comprehensive study of the structure and piezoelectric response of biodegradable polyhydroxybutyrate-based films for tissue engineering applications. Polym. J. 2022, 54, 1225–1236. 10.1038/s41428-022-00662-8.

[ref22] Meng D.; Miao C.; Liu Y.; Wang F.; Chen L.; Huang Z.; Fan X.; Gu P.; Li Q. Metabolic engineering for biosynthesis of poly(3-hydroxybutyrate-co-3-hydroxyvalerate) from glucose and propionic acid in recombinant Escherichia coli. Bioresour. Technol. 2022, 348, 126786 10.1016/j.biortech.2022.126786. 35114368

[ref23] Pracella M.; Mura C.; Galli G. Polyhydroxyalkanoate Nanocomposites with Cellulose Nanocrystals as Biodegradable Coating and Packaging Materials. ACS Appl. Nano Mater. 2021, 4, 260–270. 10.1021/acsanm.0c02585.

[ref24] Murphy C. M.; Haugh M. G.; O’Brien F. J. The effect of mean pore size on cell attachment, proliferation and migration in collagen-glycosaminoglycan scaffolds for bone tissue engineering. Biomaterials 2010, 31, 461–466. 10.1016/j.biomaterials.2009.09.063. 19819008

[ref25] Nunes-Pereira J.; Ribeiro S.; Ribeiro C.; Gombek C. J.; Gama F. M.; Gomes A. C.; Patterson D. A.; Lanceros-Méndez S. Poly(vinylidene fluoride) and copolymers as porous membranes for tissue engineering applications. Polym. Test. 2015, 44, 234–241. 10.1016/j.polymertesting.2015.05.001.

[ref26] Yuan Z.; Zhang K.; Jiao X.; Cheng Y.; Zhang Y.; Zhang P.; Zhang X.; Wen Y. A controllable local drug delivery system based on porous fibers for synergistic treatment of melanoma and promoting wound healing. Biomater. Sci. 2019, 7, 5084–5096. 10.1039/C9BM01045A. 31565707

[ref27] Lee Y.; Park S.; Kim S. I.; Lee K.; Ryu W. Rapidly Detachable Microneedles Using Porous Water-Soluble Layer for Ocular Drug Delivery. Adv. Mater.Technol. 2020, 5, 1901145 10.1002/admt.201901145.

[ref28] Reizabal A.; Brito-Pereira R.; Fernandes M. M.; Castro N.; Correia V.; Ribeiro C.; Costa C. M.; Perez L.; Vilas J. L.; Lanceros-Méndez S. Silk fibroin magnetoactive nanocomposite films and membranes for dynamic bone tissue engineering strategies. Materialia 2020, 12, 100709 10.1016/j.mtla.2020.100709.

[ref29] Liang X.; Matyushov A.; Hayes P.; Schell V.; Dong C.; Chen H.; He Y.; Will-Cole A.; Quandt E.; Martins P.; McCord J.; Medarde M.; Lanceros-Mendez S.; Van Dijken S.; Sun N. X.; Sort J. Roadmap on Magnetoelectric Materials and Devices. IEEE Trans. Magn. 2021, 57, 9446997 10.1109/TMAG.2021.3086635.

[ref30] Wei Y.; Han B.; Hu X.; Lin Y.; Wang X.; Deng X. Synthesis of Fe_3_O_4_ nanoparticles and their magnetic properties. Procedia Eng. 2012, 27, 632–637. 10.1016/j.proeng.2011.12.498.

[ref31] Díaz E.; Valle M. B.; Ribeiro S.; Lanceros-Mendez S.; Barandiarán J. M. A new approach for the fabrication of cytocompatible PLLA-magnetite nanoparticle composite scaffolds. Int. J. Mol. Sci. 2019, 20, 4664 10.3390/ijms20194664. 31547060 PMC6801398

[ref32] Sencadas V.; Ribeiro C.; Bdikin I.; Kholkin A.; Lanceros-Mendez S. Local piezoelectric response of single poly (vinylidene fluoride) electrospun fibers. Phys. Status Solidi A 2012, 209, 2605–2609. 10.1002/pssa.201228136.

[ref33] Soergel E. Piezoresponse force microscopy (PFM). J. Phys. D: Appl. Phys. 2011, 44, 464003 10.1088/0022-3727/44/46/464003.

[ref34] Gruverman A.; Kholkin A.; Kingon A.; Tokumoto H. Asymmetric nanoscale switching in ferroelectric thin films by scanning force microscopy. Appl. Phys. Lett. 2001, 78, 2751–2753. 10.1063/1.1366644.

[ref35] da Silva A. C.; Higgins M. J.; Córdoba de Torresi S. I. The effect of nanoscale surface electrical properties of partially biodegradable PEDOT-co-PDLLA conducting polymers on protein adhesion investigated by atomic force microscopy. Mater. Sci. Eng., C 2019, 99, 468–478. 10.1016/j.msec.2019.01.103. 30889721

[ref36] Nunes-Pereira J.; Martins P.; Cardoso V. F.; Costa C. M.; Lanceros-Méndez S. A green solvent strategy for the development of piezoelectric poly(vinylidene fluoride-trifluoroethylene) films for sensors and actuators applications. Mater. Des. 2016, 104, 183–189. 10.1016/j.matdes.2016.05.023.

[ref37] Kekuda D.; Lin H. S.; Chyi Wu M.; Huang J. S.; Ho K. C.; Chu C. W. The effect of solvent induced crystallinity of polymer layer on poly(3-hexylthiophene)/C70 bilayer solar cells. Sol. Energy Mater. Sol. Cells 2011, 95, 419–422. 10.1016/j.solmat.2010.05.055.

[ref38] Zhao M.; Yang Z.; Zhu D.; Jin X.; Huang D. Influence of the fabrication technique on the porous size of the polymer nanoporous antireflection coatings. J. Opt. Soc. Am. B 2005, 22, 1330–1334. 10.1364/JOSAB.22.001330.

[ref39] Ji Y.; Zhang H.; Ru J.; Wang F.; Xu M.; Zhou Q.; Stanikzai H.; Yerlan I.; Xu Z.; Niu Y.; Wei J. Creating micro-submicro structure and grafting hydroxyl group on PEEK by femtosecond laser and hydroxylation to synergistically activate cellular response. Mater. Des. 2021, 199, 109413 10.1016/j.matdes.2020.109413.

[ref40] Robledo-Taboada L. H.; Jiménez-Jarquín J. F.; Chiñas-Castillo F.; Méndez-Blas A.; Camacho-López S.; Serrano-de la Rosa L. E.; Caballero-Caballero M.; Alavez-Ramirez R.; Bartolo-Alemán M. H.; Enriquez-Porras E. N. Tribological performance of porous silicon hydrophobic and hydrophilic surfaces. J. Mater. Res. Technol. 2022, 19, 3942–3953. 10.1016/j.jmrt.2022.06.094.

[ref41] Carli L. N.; Crespo J. S.; Mauler R. S. PHBV nanocomposites based on organomodified montmorillonite and halloysite: The effect of clay type on the morphology and thermal and mechanical properties. Composites, Part A 2011, 42, 1601–1608. 10.1016/j.compositesa.2011.07.007.

[ref42] Wellen R. M. R.; Rabello M. S.; Araujo I. C.; Fechine G. J. M.; Canedo E. L. Melting and crystallization of poly(3-hydroxybutyrate): Effect of heating/cooling rates on phase transformation. Polimeros 2015, 25, 296–304. 10.1590/0104-1428.1961.

[ref43] El-Hadi A.; Schnabel R.; Straube E.; Müller G.; Henning S. Correlation between degree of crystallinity, morphology, glass temperature, mechanical properties and biodegradation of poly (3-hydroxyalkanoate) PHAs and their blends. Polym. Test. 2002, 21, 665–674. 10.1016/S0142-9418(01)00142-8.

[ref44] Aqida S.; Ghazali M.; Hashim J. Effect of porosity on mechanical properties of metal matrix composite: an overview. J. Teknol. 2004, 40, 17–32. 10.11113/jt.v40.395.

[ref45] Al-Maharma A. Y.; Patil S. P.; Markert B. Effects of porosity on the mechanical properties of additively manufactured components: a critical review. Mater. Res. Express 2020, 7, 122001 10.1088/2053-1591/abcc5d.

[ref46] Fukada E.; Ando Y. Piezoelectric properties of poly-β-hydroxybutyrate and copolymers of β-hydroxybutyrate and β-hydroxyvalerate. Int. J. Biol. Macromol. 1986, 8, 361–366. 10.1016/0141-8130(86)90056-5.

[ref47] Eaton P.; West P. Atomic Force Microscopy, 2010; ISBN 9780199570454; pp 1–256.

[ref48] Liu Y.; Zhang Y.; Chow M. J.; Chen Q. N.; Li J. Biological ferroelectricity uncovered in aortic walls by piezoresponse force microscopy. Phys. Rev. Lett. 2012, 108, 078103 10.1103/PhysRevLett.108.078103. 22401260 PMC3499944

[ref49] Gruverman A.; Rodriguez B. J.; Nemanich R. J.; Kingon A. I. Nanoscale observation of photoinduced domain pinning and investigation of imprint behavior in ferroelectric thin films. J. Appl. Phys. 2002, 92, 2734–2739. 10.1063/1.1497698.

[ref50] Ferri A.; Saitzek S.; Da Costa A.; Desfeux R.; Leclerc G.; Bouregba R.; Poullain G. Thickness dependence of the nanoscale piezoelectric properties measured by piezoresponse force microscopy on (1 1 1)-oriented PLZT 10/40/60 thin films. Surf. Sci. 2008, 602, 1987–1992. 10.1016/j.susc.2008.04.001.

[ref51] Jiang P.; Lu J.; Li K.; Chen X.; Dan R. Research on hydrophobicity of electrospun Fe_3_O_4_/PVDF nanofiber membranes under different preparation conditions. Fullerenes, Nanotubes, Carbon Nanostruct. 2020, 28, 381–386. 10.1080/1536383X.2019.1687453.

[ref52] Huang Z. H.; Zhang X.; Wang Y. X.; Sun J. Y.; Zhang H.; Liu W. L.; Li M. P.; Ma X. H.; Xu Z. L. Fe_3_O_4_/PVDF catalytic membrane treatment organic wastewater with simultaneously improved permeability, catalytic property and anti-fouling. Environ. Res. 2020, 187, 109617 10.1016/j.envres.2020.109617. 32445946

[ref53] Marques-Almeida T.; Cardoso V. F.; Ribeiro S.; Gama F. M.; Ribeiro C.; Lanceros-Mendez S. Tuning Myoblast and Preosteoblast Cell Adhesion Site, Orientation, and Elongation through Electroactive Micropatterned Scaffolds. ACS Appl. Bio Mater. 2019, 2, 1591–1602. 10.1021/acsabm.9b00020. 35026893

[ref54] Gasmi S.; Hassan M. K.; Luyt A. S. Crystallization and dielectric behavior of PLA and PHBV in PLA/PHBV blends and PLA/PHBV/TIO 2 nanocomposites. eXPRESS Polym. Lett. 2019, 13, 199–212. 10.3144/expresspolymlett.2019.16.

[ref55] Li Z.; Reimer C.; Wang T.; Mohanty A. K.; Misra M. Thermal and mechanical properties of the biocomposites of Miscanthus biocarbon and poly(3-hydroxybutyrate-co-3-hydroxyvalerate) (PHBV). Polymer 2020, 12, 1300 10.3390/polym12061300. PMC7362254 32517200

[ref56] Brito-Pereira R.; Ribeiro C.; Peřinka N.; Lanceros-Mendez S.; Martins P. Reconfigurable 3D-printable magnets with improved maximum energy product. J. Mater. Chem. C 2020, 8, 952–958. 10.1039/C9TC06072C.

[ref57] Castro N.; Ribeiro S.; Fernandes M. M.; Ribeiro C.; Cardoso V.; Correia V.; Minguez R.; Lanceros-Mendez S. Physically Active Bioreactors for Tissue Engineering Applications. Adv. Biosyst. 2020, 4, 2000125 10.1002/adbi.202000125. 32924326

[ref58] Martins P.; Lanceros-Méndez S. Polymer-based magnetoelectric materials: To be or not to be. Appl. Mater. Today 2019, 15, 558–561. 10.1016/j.apmt.2019.04.004.

