# Supplementary material for: Piezoelectric and Magnetically Responsive Biodegradable Composites with Tailored Porous Morphology for Biotechnological Applications
Source: ACS Appl Polym Mater. 2022 Nov 9;4(12):8750–63. doi: 10.1021/acsapm.2c01114 (PMC9778034; doi:10.1021/acsapm.2c01114)
Supplement: Supplementary file 1 — ap2c01114_si_001.pdf [file ap2c01114_si_001.pdf]

Supporting information

Piezoelectric and magnetically responsive biodegradable composites with tailored porous morphology for biotechnological applications

Teresa Marques-Almeida[1,2], Vitor Correia[3,4], Eduardo Fernández Martín[5], Ander García Díez[4], Clarisse Ribeiro[1,2,*], Senentxu Lanceros-Mendez[5,6]

[1]Physics Centre of Minho and Porto Universities (CF-UM-UP), University of Minho, 4710-057 Braga, Portugal
[2]LaPMET - Laboratory of Physics for Materials and Emergent Technologies, University of Minho, 4710-057 Braga, Portugal
[3]CMEMS – UMinho, University of Minho, 4800-058, Guimarães, Portugal
[4]LABBELS –Associate Laboratory, Braga, Guimarães, Portugal
[5]BCMaterials, Basque Centre for Materials and Applications, UPV/EHU Science Park, Leioa, 48940, Spain
[6]IKERBASQUE, Basque Foundation for Science, Bilbao, 48009, Spain

*cribeiro@fisica.uminho.pt

Table S1. Weight percentage of Fe in the different composite films obtained through EDS analysis.

	**Fe% Weight**
**PHBV+ 5% Fe_3O_4**	4.3
**PHBV+ 10% Fe_3O_4**	9.6
**PHBV+ 20% Fe_3O_4**	18.6
**PHBV+ 10% Fe_3O_4 (CF/DMF)**	9.0
